# Mature Gastric Teratoma: The Mixed Exogastric and Endogastric Variety 

**Published:** 2011-07-30

**Authors:** Lubna Ijaz, Imran Aslam, Afzal Sheikh, Bilal Mirza

**Affiliations:** Department of Pediatric Surgery, The Children's Hospital and the Institute of Child Health Lahore, Pakistan

**Keywords:** Gastric teratoma, Endogastric tumor, Exogastric tumour

## Abstract

Gastric teratomas are extremely rare tumors. A 15-day-old neonate presented with abdominal mass. Ultrasound of abdomen showed mixed echogenicity lesion. CT scan showed a mass with solid and cystic components and internal calcifications. At operation a tumor arising from the posterior wall of the stomach found. It was exogastric as well as endogastric in location. Biopsy report was suggestive of mature teratoma.

## INTRODUCTION

Gastric teratomas are extremely rare tumors. They mostly present as exogastric growths, but can occur as a mix of exogastric as well as endogastric extension. They are classified into mature and immature teratomas based upon presence and differentiation of neuroglial tissue. Mature gastric teratomas are benign and have good prognosis after complete surgical excision [1]. A neonate with mature gastric teratoma of mixed variety is being reported. 

## CASE REPORT

A 15-day-old male baby presented with a palpable abdominal mass in left upper abdomen noted by parents since birth. There were no other complaints besides occasional non-bilious vomiting. Ultrasound of the abdomen showed a mixed echogenicity mass with solid and cystic areas. Computed tomography (CT) scan revealed a solid and cystic mass with internal calcifications suggestive of teratoma (Fig. 1). All the baseline laboratory investigations were within normal limits. Alpha fetoprotein (AFP) was 21 i.u.

**Figure:**
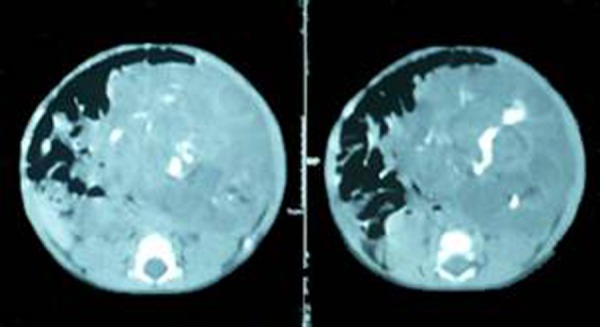
Figure 1: CT scan showing solid and cystic components with internal calcifications.

At exploration, a huge mass arising from the posterior wall of the stomach was found (Fig. 2). It was dissected out from the surrounding tissues. The stomach was palpated, before excision of the mass, which revealed a small extension of the mass into the gastric lumen (Fig.3). The mass was excised in toto with 0.5 cm margins of the gastric wall all-around. The histopathology described the mass (mature gastric teratoma) as composed of adipose tissue, stratified squamous epithelium, dermal appendages, hair follicles, gut mucosa, and mature glial tissue. The margins were tumor free. The follow up remained uneventful.

**Figure:**
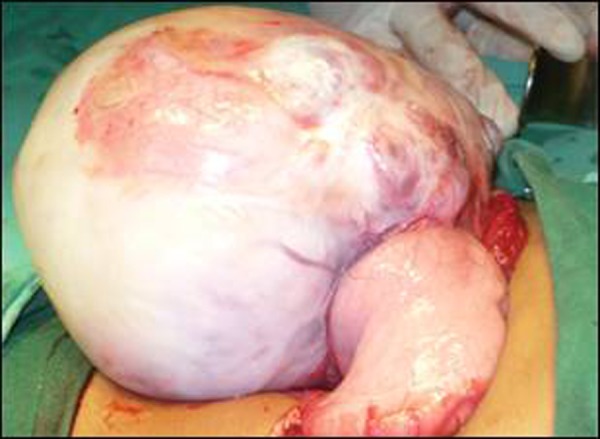
Figure 2: Mass arising from the posterior gastric wall.

**Figure:**
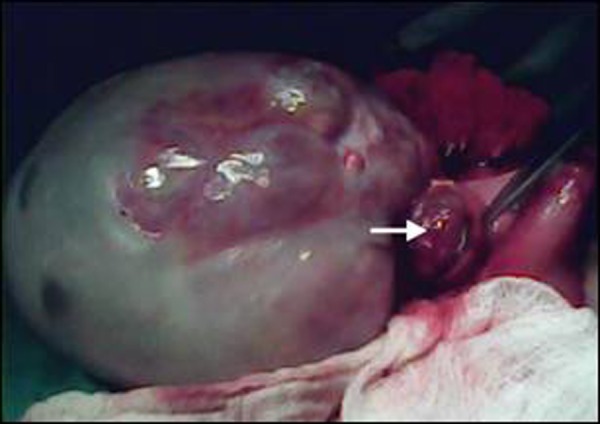
Figure 3: The posterior gastric wall was opened to show endogastric component (arrow).

## DISCUSSION

 The word teratoma is derived from Greek word “teratomas” meaning “monstrous growth”. Generally, they are composed of tissue related to all the germinal layers. Gastric teratoma was initially described by Eustermann et al in 1922. It is extremely rare tumor and forms 1% of all the teratomas in the body. Gastric teratomas may be mature and immature, based on the presence of immature glial tissue. Mature gastric teratomas contain mature glial tissue along with other derivatives of all germinal layers as found in our case. Mature gastric teratomas are considered benign tumors, whereas, the malignant potential is present in immature gastric teratomas [1, 2, 3, 4].

Majority of gastric teratomas are exogastric (>60%); endogastric growths are present in 30% of cases. Mixed exogastric and endogastric growths are rare [1]. In our case the main component of the mass was exogastric (90%) whereas a small proportion was endogastric which was detected by palpating the gastric lumen before its excision.

The main clinical features are abdominal distension, a palpable mass in the epigastrium and left abdomen, vomiting, and respiratory distress. In case of endogastric component there may be additional upper alimentary tract bleeding (hemetemesis and melena), and pain abdomen [1, 2, 3]. In our case the main presentation was a palpable abdominal mass with occasional emesis.

Abdominal radiograph, ultrasonography, CT/MRI, and endoscopy are important diagnostic tools. In most of the cases the preoperative diagnosis of gastric teratomas is difficult. Our preoperative diagnosis was gastric teratoma based upon our previous experience of dealing with immature gastric teratoma, age of the patient, and location in relation to the stomach.

Gastroscopy in case of endogastric component may aid in the preoperative diagnosis. Complete excision with tumor free margins is the goal. Long term follow up for recurrence is important. Recurrence in a case of completely excised mature gastric teratoma is seldom reported [4].

## Footnotes

**Source of Support:** Nil

**Conflict of Interest:** None declared
